# The risk reduction effect of sediment production rate by understory coverage rate in granite area mountain forest

**DOI:** 10.1038/s41598-021-93906-1

**Published:** 2021-07-13

**Authors:** Toshiaki Mizuno, Nagahiro Kojima, Satoshi Asano

**Affiliations:** 1 Lake Biwa Environmental Research Institute, 5-34, Yanagasaki, Otsu, Shiga 520-0022 Japan; 2 Research Institute for Humanity and Nature, 457-4 Motoyama, Kamigamo, Kita-ku, Kyoto, 603-8047 Japan; 3 Graduate School of Global Environmental Studies, Kyoto University, Yoshida-Honmachi, Sakyo-Ku, Kyoto, 606-8501 Japan

**Keywords:** Climate change, Hydrology, Limnology, Agroecology, Biogeography, Climate-change ecology, Conservation biology, Ecological modelling, Ecosystem ecology, Ecosystem services, Environmental economics, Forest ecology, Forestry, Freshwater ecology, Grassland ecology, Restoration ecology, Environmental impact, Ecology, Ecology, Environmental sciences, Environmental social sciences, Hydrology, Limnology, Natural hazards

## Abstract

Ecosystem-based disaster risk reduction (Eco-DRR) is an important concept to the adaption of climate change for a sustainable life. In Japan, it is anticipated that damages caused by sediment production will be increased as the intensity and amount of rainfall are increased by climate change. Thus, we need to know the Eco-DRR effect of the forest for planning sustainable land use by evidence-based data. In this study, we focused on the relationship between sediment production rate and the understory coverage rate of a low mountain forest in the granite area. From the results of the field survey and statistical meta-analysis, the sediment production rate was reduced by 97% in granite area mountain forest when the understory coverage rate was 60% or more compared to when less than 30% by evidence-based data. Accordingly, we found that it will be necessary to keep forests with an understory coverage rate of 60% or more when considering the risk-reducing effect of sediment disaster in granite area mountain forests for the adaption of climate change.

## Introduction

Ecosystem-based disaster risk reduction (Eco-DRR) is an important concept to the adaption of climate change for a sustainable life^1^. Thus, we need to know the Eco-DRR effect of the forest for planning sustainable land use by evidence-based data^2^. In Japan, it is anticipated that damages caused by sediment production will be increased as the intensity and amount of rainfall are increased by climate change^3^. Particularly, in the forest area, the extreme run-off will take place when over total rainfall 90 (mm) in low mountain forests in granite areas^4^. In fact, associated with Typhoon No. 19 (Hagibis) in 2019, the total rainfall from October 10th to 13th was over 1000 (mm) in Hakone Town, Kanagawa Prefecture, and exceeded 500 (mm) per 24 h at 17 sites in eastern Japan, which was possibly the highest rainfall since 1982. Therefore, the typhoon caused 952 sediment disasters^5^. Furthermore, recently, in the forest areas of Japan, the decrease of understory coverage rate by overgrazing deer has become a serious problem. Moreover, it is suspected that deer overgrazing damage may have contributed to the sediment disaster in mountain forests^6^.

However, there were few evidence-based research reports about the Eco-DRR effect on the understory coverage of forests in Japan^7^. Thus, we should show evaluated quantitatively the sediment disaster risk reduction effect in granite area mountain forest by evidence-based data. In this study, we focused on the relationship between sediment production rate and understory coverage rate in a low mountain forest in the granite area in Japan.

## Results

As a result of the field observation, in the understory coverage rate 0–30%, the range of annual maximum 72-h rainfall was 133–540 (mm), the average value was 322 (mm), the median was 343(mm), and the range of annual sediment production was 60.1–3779.5 (m^3^/km^2^/year), with an average of 1247.2 (m^3^/km^2^/year) and a median of 695.7 (m^3^/km^2^/year).

In the understory coverage 30–60%, the range of annual maximum 72-h rainfall was 129–455 (mm), the average was 271 (mm), the median was 256 (mm), and the range of annual sediment production was 7.6–1192.4 (m^3^/km^2^/year), the average value was 246.3 (m^3^/km^2^/year) and the median was 161.5 (m^3^/km^2^/year).

In the understory coverage of 60–100%, the range of annual maximum 72-h rainfall was 91–200 (mm), the average was 137 (mm), the median was 133 (mm), and the range of annual sediment production was 1.3–18.5 (m^3^/km^2^/year), the average value was 7.3 (m^3^/km^2^/year), and the median value was (m^3^/km^2^/year).

**Figure 1:**
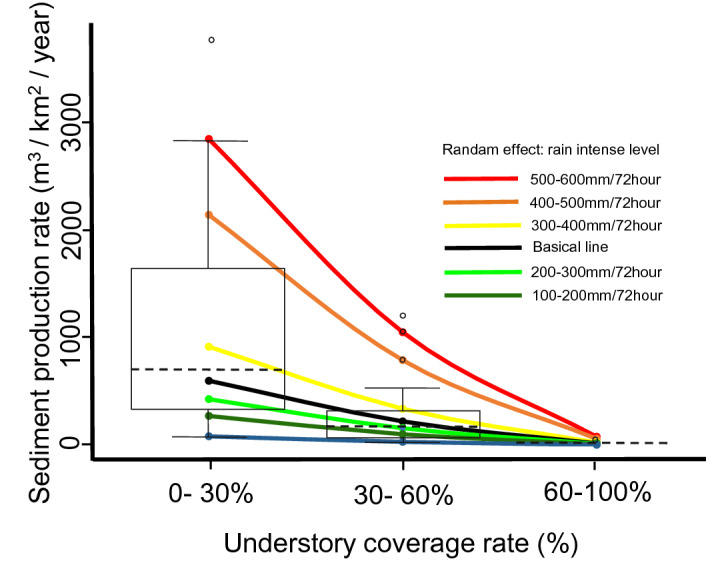
The result of regression analysis about the relationship between sediment production rate and understory coverage rate. The boxplot shows the sediment production per 1 km^2^ on the surface of each understory coverage rate of 0–30%, 30–60%, 60–100%. The graph was drawn by T. Mizuno using Microsoft PowerPoint. The boxplot was based on the graphic of the calculated results by R Commander version 2.7–1^25,26^ and the line by using the calculated results of the Poisson regression analysis of lme4 package version 1.1–26^21^.

Next, we were using the observed evidence data, and we set the understory coverage rate as an explanatory variable, the annual sediment production per 1.0 (km^2^) as a dependent variable, and the maximum 72-h annual rainfall as a random effect. Finally, we analyzed by Poisson mixed-effect regression model. As a result, the intercept for the fixed effect was 6.39, the coefficient for the understory coverage 30–60% was − 1.00 (Pr: 0.000 < 0.001), and the coefficient for the understory coverage 60–100% was -3.60 (Pr: 0.000 < 0.001). Regarding the random effect on the intercept by the maximum 72-h annual rainfall, 100–200 (mm) was − 1.99, 200–300 (mm) was − 0.79, 300–400 (mm) was − 0.34, 400–500 (mm) was 0.43, 500–600 (mm) was 1.28, 600–700 (mm) was 1.56 (Fig. 1).

The equation of the result of the Poisson mixed-effect regression analysis was below:

$$ {\text{Y}} = {\text{e}}^{{\left( {\left( { - 1.00} \right){\text{X}}_{{30 - 60}}  + \left( { - 3.60} \right){\text{X}}_{{60 - 100}}  + 6.39 + {\text{random}}\;{\text{effect}}_{{{\text{rain}}\;{\text{intense}}}} } \right)}} $$

X as either a 1 or a 0, all combination was below:

$$ \begin{array}{*{20}c}    {\left\{ {\begin{array}{*{20}c}    {{\text{X}}_{{30 - 60}}  = 0}  \\    {{\text{X}}_{{60 - 100}}  = 0}  \\   \end{array} } \right.} & {\left\{ {\begin{array}{*{20}c}    {{\text{X}}_{{30 - 60}}  = 1}  \\    {{\text{X}}_{{60 - 100}}  = 0}  \\   \end{array} } \right.} & {\left\{ {\begin{array}{*{20}c}    {{\text{X}}_{{30 - 60}}  = 0}  \\    {{\text{X}}_{{60 - 100}}  = 1}  \\   \end{array} } \right.}  \\   \end{array} $$

From the Poisson mixed regression analysis results, the annual sediment production per 1.0 (km^2^) was 595.9 (m^3^/km^2^/year) when the understory coverage was 0–30% without the random effects of rainfall. Second, when the understory coverage was 30–60%, the annual sediment production per 1.0 (km^2^) was 219.2 (m^3^/km^2^/year). Third, when the understory coverage was 60–100%, the annual sediment production per 1.0 (km^2^) was 16.3 (m^3^/km^2^/year). Comparing these results with the case where the understory coverage was 0–30%, the attenuation rate of the annual sediment production per 1.0 (km^2^) was 63.2% when the understory coverage was 30–60%. Furthermore, the attenuation rate of the annual sediment production per 1.0 (km^2^) was 97.3% when the understory coverage was 60–100%.

## Discussion

The mechanism by which these results were obtained was thought to be due to the surface flow of forest slopes. Surface flow on forest slopes is said to include the Horton overland flow^8^ and the saturated overland flow^9^ that occurs when the soil was saturated. The target area of this study was an area where the volume moisture content θ of the soil was approximately 0.15–0.19 (m^3^/m^3^)^10^ and there are many planted cypress forests. It has been confirmed that not only the saturated overland flow but also Horton overland flow often occurred on the forest floors in this area during rainfall^11^. And it thought that the difference in forest floor covering affects the surface runoff^12^.

The Universal Soil Loss Equation: USLE^13^ was known as a formula for predicting erosion of the USDA. USLE was a model formula with 6 variables to determine the amount of erosion. Erosion = RKLSCP: R was the rainfall factor, K was the soil erodibility factor, L was the slope length factor, S was the steepness factor. In the application to forests, C may be called the vegetation and management factor. P was the support practice factor. Kitahara^14^ examined variables in Japanese forests and noted that C and P values are most important for predicting soil erosion. The results of this study tended to be consistent with Kitahara's study that the understory coverage rate affects soil erosion.

Hattori et al.^15^ has conducted a previous study on a cypress forest with a slope of approximately 30° in the granite area of the Lake Biwa basin, which was very similar to this study. Hattori's study was compared the annual sediment production between the understory coverage rate of about 50–60% by dwarf bamboo (Sasa) in the cypress forest was about and the understory coverage rate was about 0–30%. When dwarf bamboo (Sasa) planting coverage of this forest floor was about 50–60%, the attenuation rate of the sediment production was 89.1%. This value was between the attenuation rate of 63.2% when the understory coverage was 50–60% and the attenuation rate of 97.3% when the understory coverage was 50–100%. From the results, it was confirmed that the attenuation rate estimated from the model of this study was in agreement with the results of the existing Hattori's study.

Forests are also said to have a reducing effect on landslide risk^16^. Landslide disasters correlate with the density of vegetation on mountain slopes, and landslides are reduced when the vegetation density was high^17^. In this study as well, the vegetation density was extremely high. Because there are not only the woods but also the understory coverage. Therefore, we considered that the increased understory coverage rate not only reduces the sediment production but also the risk of landslide disasters by climate change. In the future, we think that we will try to examine and analyze the understory coverage data where landslides have occurred, and we will try to quantitatively evaluate the understory coverage rate as green infrastructure for the climate change adaption.

## Methods

**Figure 2:**
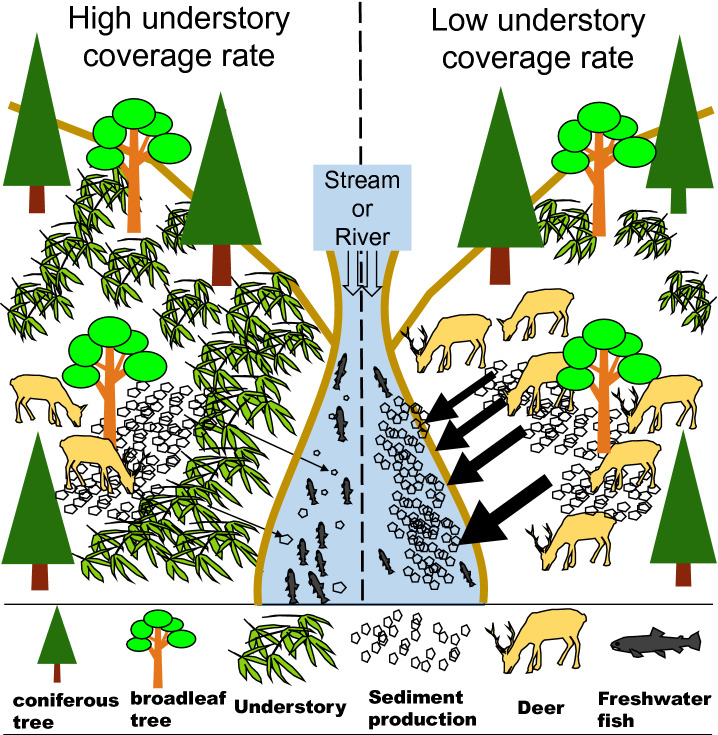
The concept ecosystem image diagram of the relationship between understory coverage rate and sediment production in the low mountain forest area. The understory disturbs sediments moving. The concept image diagram was drawn by T. Mizuno using Microsoft PowerPoint.

**Figure 3:**
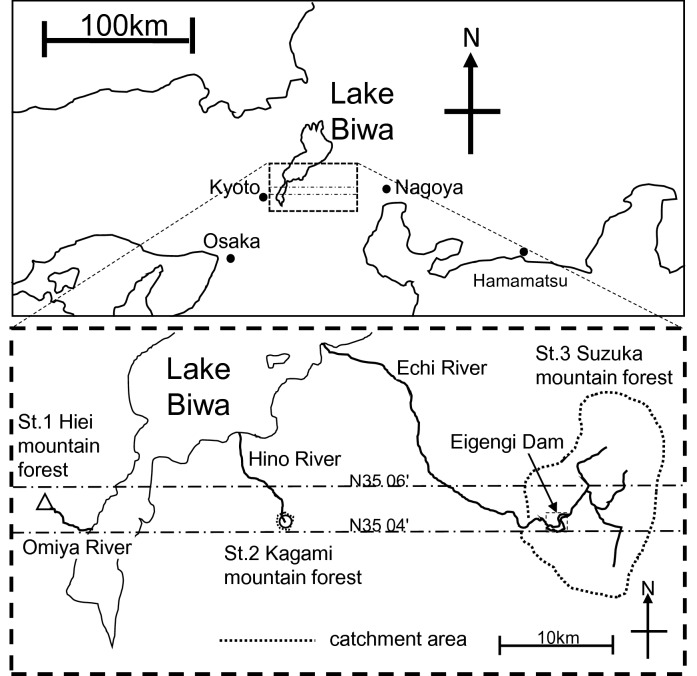
The location map of the field survey forest of the low mountain on granite are between N35 06' from N35 04', where are the basin of Lake Biwa near Kyoto in the western area of Japan. The location map was based on the Digital Topographic Map 25,000 published by the Geospatial Information Authority of Japan (https://maps.gsi.go.jp), and was edited and processed by T. Mizuno using Microsoft PowerPoint.

In this study, as shown in the conceptual image described in Fig. 2, we supposed that the cause to promote sediment production rate from forest areas to rivers is understory coverage rate decreasing. As the locations of the survey, we selected three granite low mountain forest areas in Japan; Hiei mountain, Kagami mountain, and Suzuka mountain. These places were the similar environmental conditions, forest woods, understory plants, the incline which almost less than 35°, and the latitude which is N35°04'-05' at catchments of the river which inflow to Lake Biwa. We surveyed Shiga prefecture where is almost Lake Biwa basin in the western area of Japan. The forest area of Shiga prefecture is about 200,000 ha (about 60% occupied the prefecture area). About 80,000 ha of them are the artificial forest of cedar and cypress. Most of the planted forests are of an age that can be cut down and used. The locations of the field survey, we selected three low mountain forest areas on granite area and almost the same latitude in Lake Biwa basin; Hiei mountain, Kagami mountain, and Suzuka mountain (Fig. 3).

For the sediment production rate, annual actual measurement data of sediment receiving boxes, sediment receiving weirs, and dams were used. For the understory coverage rate, the Miura method was used^18^. The almost physical flow of forest surfaces is expressed in the form of a function of exponentiation^19^. Therefore, in the meta-analysis of all field data, the Poisson regression analysis (log function) of the generalized linear mixed-effects regression model was used. The dependent variable was the sediment production rate, the explanatory variable was the understory coverage rate and the random effect was the rain intensity. Because the rainfall intensity has a large effect on the surface flow of the forest when the amount of rainfall exceeds 90 (mm) on the moist soil^4^. Therefore, assuming that the maximum annual rainfall of 72 h is strongly related to sediment movement, it was used as an index of rainfall intensity. All-year data of Hiei, Kagami, and Suzuka of the maximum annual rainfall of 72 h exceeded 90 (mm). The rainfall intensity was categorized 6 levels; 100–200 (mm), 200–300 (mm), 300–400 (mm), 400–500 (mm), 500–600 (mm) and 600–700 (mm). Each rainfall intensity was inserted as the random effect in the Poisson regression analysis model. Statistical analysis software used R^20^ and performed calculations using the lme4 package^21^.

### Detail method of field survey of Hiei mountain forest (St.1)

We carried out a field survey on the sediment production rate on a forest slope in the Hiei mountain forest owned by Enryakuji temple which was recorded world heritage. The stream order of the survey site is the zero-order basin of the Omiya River. The survey point was set in the forest of Sakamoto-Cho, Otsu City (Latitude and longitude notation; 35° 5′ 29ʺ N, 135° 50′ 10ʺ E) located in the uppermost stream of the Omiya River in the southwestern part of Shiga Prefecture. The bedrocks were mainly granite rock and the soil was brown forest soil. The altitude was about 760 (m), the slope direction was east, and the slope was 32°–35°. The main forest wood was the Japanese cypress (*Chamaecyparis obtusa*), which was about 100 years old, and the forest floor was relatively bright with moderate forest density. Besides, dwarf bamboo flourished on the forest floor of the study site and nearby forests until around 2005. At present, there are many areas where understory vegetation has disappeared due to deer feeding damage. At the survey point, the surface of the forest floor had become bare. On the forest slope where the understory vegetation had disappeared, we made a 5.0 (m) × 5.0 (m) survey area surrounded by a protective fence with a height of about 2.0 (m) to prevent deer feeding damage. At the lower end of the survey area, five sediment receiving boxes with a width of 25 (cm) and a height of 15 (cm) were installed at intervals of about 1.0 (m) along the contour lines. The survey started in June 2015, and the samples captured in the sediment receiving box were collected approximately once every two to four weeks, and after heavy rain appropriately. The collected sediment sample was air-dried, dried at 70 °C for 24 h or more, fractionated into sediment and litter, and the weight of each was measured. The sediment production rate was converted with a specific gravity of 1.8 (tons) per 1.0 (m^3^). As for the understory coverage rate, vegetation growth, litter, sediment, and gravel were evaluated by the point-counting by the Miura method^18^ in a range of 50 (cm) × 50 (cm) above each sediment receiving a box every autumn. Also, we checked the vegetation overgrowth around the field survey area. As the rainfall data used in the analysis, the observation data (observatory name: Hiei) closest to the survey site was used from the water quality hydrology database of the Ministry of Land, Infrastructure, Transport, and Tourism. The boxplot of the annual sediment production rate (m^3^/km^2^/year) was made by using soft-wares R3.6.1^20^.

### Detail method of field survey of Kagami mountain (St.2)

We collected data about the sediment production rate of the forest with 60% or more of the understory coverage rate in the Kagami mountain forest where no deer has been confirmed. The stream order of the survey site was a 0–3 order basin within the catchment area of the Hino River. The investigation point of sediment outflow from the forest was conducted at the forest mountain stream in Oshinohara, Yasu City (Latitude and longitude notation; 35° 4′ 2ʺ N, 136° 4′ 3ʺ E). The bedrock is granite, and the soil is brown forest soil. The catchment area of the study site is 20.0 ha, the altitude is about 150–280 (m), the slope of the mountain stream is north, and the slope of the mountain stream is about 11°. The main forest wood was the Japanese cypress (*Chamaecyparis obtusa*) and deciduous broad-leaved trees such as oak. No deer feeding damage to adult trees and understory was observed high density in the survey area. Mainly understory is the fern plant (*Gleichenia japonica*). Now the understory coverage rate is 60% or more anywhere. Sediment and litter that flowed out of the forest were collected from the upper part of the concrete weir (2.4 (m) wide × 1.2 (m) tall) installed at the downstream end of the survey site. We collected approximately once every two to four weeks after heavy rain in five years from 2015 to 2019. The collected sediment sample was air-dried for about 1 week, then dried at 70 °C for 24 h or more, and the weight of gravel and litter was measured. The boxplot of the annual sediment production rate (m^3^/km^2^/year) was made by using soft-wares R3.6.1^20^.

### Detail method of data collection of Suzuka mountain (St.3)

We collected data about the sediment production rate of the forest with both cases under 30% and 30%-60% of the understory coverage rate in the Suzuka mountain by using the Eigenji dam annual sediment deposit data. The elevation of the Eigenji Dam dam is 274 (m), and the maximum elevation of the catchment area of the Eigenji Dam is 1247 (m). The Eigenji dam is the stream order which is a 0–6 order basin within the catchment area of the Echi River. The Eigenji Dam was built in 1973 on the Echi River in Higashi-Omi City, Shiga Prefecture (Latitude and longitude notation; 35° 4′ 35ʺ N, 136° 20′ 7ʺ E), the catchment area is 131.5 km^2^. The catchment area is almost the forest area of the Suzuka mountain. The main bedrock of the Eigenji dam is granite, and the main bedrocks of the catchment are granite and sedimentary rock. The soil is brown forest soil. The slope of the watershed area is 10–20°. The report of Shiga prefecture referred to the damage caused by overgrazing by deer began to increase around 2010^22,23^. In 2011, a large decrease in understory was confirmed in the entire watershed of the Eigenji Dam^24^. The boxplot of the annual sediment deposition (m^3^/km^2^/year) was made by dividing the period's case 1 is when a 30–60% understory coverage rate from 1982 to 2009 and case 2 is when under 30% understory coverage rate from 2010 to 2015 by using soft-wares R3.6.1^20^.

### Detail explains of the random effect of the equation of meta-analysis

The Poisson regression analysis (log function) of the generalized linear mixed-effects regression model was used. The dependent variable was the sediment production rate, the explanatory variable was the understory coverage rate and the random effect was the rainfall intensity. The rain intensity was categorized 6 levels; 100–200 (mm), 200–300 (mm), 300–400 (mm), 400–500 (mm), 500–600 (mm) and 600–700 (mm). Each rain intensity was inserted as a random effect in the Poisson mixed-effect regression analysis model. Statistical analysis software used R and performed calculations using the lme4 package^21^.
